# LIVING LIFE TO THE FULL 55-PLUS: A COMMUNITY-BASED INTERVENTION FOR REDUCING DEPRESSION AND ANXIETY

**DOI:** 10.1093/geroni/igz038.1887

**Published:** 2019-11-08

**Authors:** Nasreen Khatri, Stephen Perkovic, Tara Faghani Hamadani

**Affiliations:** 1 Rotman Research Institute, Baycrest, Toronto, Ontario, Canada; 2 York University, Toronto, Ontario, Canada; 3 Dalla Lana School of Public Health, University of Toronto, Toronto, Ontario, Canada

## Abstract

Living Life to the Full 55+ (LLTTF) was an 8-week, 12-hour, community educational program based on CBT principles. The program aim was to teach participant skills and techniques to cope effectively with life stress. Participants self-referred to the program by responding to advertisements or were otherwise referred to the program by local community centres. Following the promising results of the pilot study, a follow-up empirical study was conducted to assess the impact of the program on clinical measures and other notable measures over time. Study participants (N = 514) were recruited at partner sites hosting the program. Demographic and other data was collected to assess the impact of the program. Measures collected to assess the impact of the program were the Beck Depression Inventory (BDI-II), Beck Anxiety Inventory (BAI), Warwick-Edinburgh Mental Well-being Scale (WEMWBS), and a brief measure of loneliness/social connectedness. Participant data was collected pre/post-intervention, and at post-course follow-up periods (3/6/9-months). Preliminary repeated-measures ANOVA analyses found statistically significant changes in depression, anxiety, mental well-being, and loneliness scores, from pre- to post-intervention. Participant’ depression, anxiety, and loneliness scores significantly reduced from pre- to post-course. Participants also experienced a significant improvement in mental well-being from pre- to post-course. Observed decreases in loneliness and depression did not significantly change from post-course to any of the follow-up periods. Results suggest the viability of a nonclinical program for addressing depression and loneliness in older individuals, and have implications for community-based strategies attempting to address mental health issues in older Canadians

